# Cardiovascular magnetic resonance risk stratification in patients with clinically suspected myocarditis

**DOI:** 10.1186/1532-429X-16-14

**Published:** 2014-01-26

**Authors:** Julia Schumm, Simon Greulich, Anja Wagner, Stefan Grün, Peter Ong, Kerstin Bentz, Karin Klingel, Reinhard Kandolf, Oliver Bruder, Steffen Schneider, Udo Sechtem, Heiko Mahrholdt

**Affiliations:** 1 Department of Cardiology, Robert Bosch Medical Center, Stuttgart, Germany; 2 Comprehensive Cardiology of Stamford and Greenwich, Stamford, CT, USA; 3 Department of Molecular Pathology, University of Tübingen, Tübingen, Germany; 4 Institut für Herzinfarktforschung Ruhr, Essen, Germany

**Keywords:** Cardiovascular magnetic resonance, Risk stratification, Myocarditis, Outcome

## Abstract

**Background:**

The diagnosis of myocarditis is challenging due to its varying clinical presentation. Since myocarditis can be associated with significant 5-year mortality, and postmortem data show myocarditis in almost 10% of all adults suffering sudden cardiac death, individual risk stratification for patients with suspected myocarditis is of great clinical interest. We sought to demonstrate that patients with clinically suspected myocarditis and a normal cardiovascular magnetic resonance (CMR) according to our definition have a good prognosis, independent of their clinical symptoms and other findings.

**Methods:**

Prospective clinical long-term follow-up of consecutive patients undergoing CMR for work-up of clinically suspected myocarditis at our institution in 2007-2008.

**Results:**

Follow-up was available for n = 405 patients (all-comers, 54.8% inpatients, 38% outpatient referrals from cardiologists). Median follow-up time was 1591 days. CMR diagnosis was “myocarditis” in 28.8%, “normal” in 55.6% and “other pathology” in 15.6%. Normal CMR was defined as normal left ventricular (LV) volumes and normal left ventricular ejection fraction (LV-EF) in the absence of late Gadolinium Enhancement (LGE). The overall mortality was 3.2%. There were seven cardiac deaths during follow-up, in addition one aborted SCD and two patients had appropriate internal cardioverter defibrillator (ICD) shocks – all of these occurred in patients with abnormal CMR. Kaplan-Meier analysis with log-rank test showed significant difference for major adverse cardiac events (cardiac death, sudden cardiac death (SCD), ICD discharge, aborted SCD) between patients with normal and abnormal CMR (p = 0.0003).

**Conclusion:**

In our unselected population of consecutive patients referred for CMR work-up of clinically suspected myocarditis, patients with normal CMR have a good prognosis independent of their clinical symptoms and other findings.

## Background

The diagnosis of myocarditis is challenging due to its varying clinical presentation. Especially in patients with non-specific or mild symptoms it can be difficult to make or to exclude. This is a major clinical problem, since patients with severe forms of myocarditis were recently shown to have a 5-year mortality of almost 20% [1]. Postmortem examinations show myocarditis in almost 10% of all adults suffering sudden cardiac death (SCD) [2]. Therefore, individual risk stratification for patients with suspected myocarditis is of great clinical interest.

Cardiovascular magnetic resonance (CMR) offers important incremental prognostic information in a variety of cardiac diseases like different forms of cardiomyopathies or coronary artery disease [3-5], and also shows promise for risk stratification in inflammatory myocardial disease [1,6].

Consequently, we hypothesized that patients referred for CMR work-up of suspected myocarditis can be risk stratified on the basis of routine CMR parameters, such as ventricular size and function, as well as the presence of late gadolinium enhancement (LGE). In particular, we sought to demonstrate that patients with clinically suspected myocarditis that have a normal CMR according to our definition have a good prognosis, independent of their clinical symptoms and other findings.

## Methods

### Patient population

**Figure 1:**
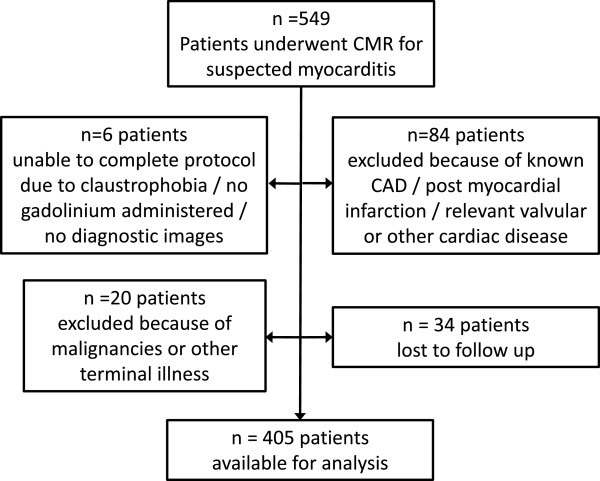
Flow chart visualizing the derivation of the study population.

We prospectively followed 405 consecutive patients who underwent CMR for work-up of clinically suspected myocarditis at our institution between 01.01.2007 and 03.07.2008. Thus, the main inclusion criteria was a clinical suspicion for myocarditis by each treating physician that was strong enough to refer the patient for CMR work-up of suspected myocarditis, reflecting a real world clinical routine population. Patients with previously known coronary artery disease (CAD), post myocardial infarction, or relevant valvular diseases, as well as patients with previously known malignancies, other terminal illness, or non-diagnostic images were excluded (Figure 1). The local ethics committee (University of Tübingen, Germany) approved data collection and management and each patient gave informed consent. Some of the patients (n = 25) were part of a previous report [1]. In the current cohort, endomyocardial biopsy (EMB) was not routine part of the study protocol as in previous cohorts [1,7], but was only performed if clinically indicated [8]. Histopathological analysis and immunohistology were used to evaluate EMB samples as described previously [1,7,9].

### CMR protocol and analysis

Electrocardiogram (ECG) gated CMR imaging was performed in breath-hold using a 1.5 T Magnetom Sonata (Siemens-Healthcare, Germany) in line with recommendations of the Society of CMR (SCMR) and the European Society of Cardiology (ESC) Working Group EuroCMR, respectively [10]. Both cine and LGE short axis CMR images were prescribed every 10 mm (slice thickness 6 mm) from base to apex. In-plane resolution was typically 1.2×1.8 mm. Cine CMR was performed using a steady-state-free-precession-sequence. LGE images were acquired on average 5–10 minutes after contrast administration using segmented inversion recovery gradient echo sequences (IR-GRE) [11] constantly adjusting inversion time to null normal myocardium [12]. The contrast dose (gadodiamide or gadopentetate-dimeglumine) was 0.15 mmol/kg.

Cine and contrast images were evaluated by two experienced observers as described elsewhere [7]. In brief; endocardial and epicardial borders were outlined on the short-axis cine images, papillary muscles were treated as myocardium and included in the analysis. Volumes and ejection-fraction were derived by summation of epicardial and endocardial contours. The extent of LGE was assessed using the Siemens Argus analysis software package. The extent of LGE was assessed on the short-axis contrast images with the use of an image intensity level ± 2 SD above the mean of remote myocardium to define LGE [1,7,9].

### Clinical follow-up, variables, endpoints and definitions

All variables were collected directly from patients, and/or medical records except CMR parameters, which were evaluated as described above. Most variables are self-explanatory; all others are defined below.

Clinical follow-up was performed using a standardized telephone questionnaire at the earliest three years after initial presentation for CMR. In case of a suspected event, all necessary medical records were obtained and reviewed in blinded fashion by some of the authors (J.S., S.G., H.M.) acting as an end point committee.

The predefined primary endpoints were major adverse cardiac events, including cardiac death, sudden cardiac death, aborted sudden cardiac death, and appropriate ICD discharge. The secondary endpoint was defined as a composite of primary endpoint and hospitalization for heart failure. In detail, the following definitions were used:

Major adverse cardiac event: All cardiac death, including SCD, and aborted SCD.

Cardiac death: death from all cardiac causes.

SCD: unexpected arrest of presumed cardiac origin within one hour after onset of any symptoms that could be interpreted as being cardiac in origin.

Aborted sudden cardiac death was defined as resuscitation after cardiac arrest in a patient who remained alive 28 days later.

Defibrillator discharges considered appropriate included automatic defibrillation shocks triggered by ventricular tachycardia or fibrillation and documented by stored intracardiac electrocardiographic or cycle-length data.

Hospitalization for heart failure: Hospitalization as an in-patient >24 h, and heart failure as primary diagnosis according to the hospitals final report.

A normal CMR was defined as left ventricular ejection fraction (LV-EF) ≥ 60%, AND left ventricular end-diastolic volume (LV-EDV) ≤ 180 ml, AND no LGE present.

### Statistical analysis

Absolute numbers, percentages and medians (with quartiles) were computed to describe the patient population. Categorical variables were compared by chi-square test or Fisher exact test as appropriate; continuous parameters by using Wilcoxon rank-sum test. Kaplan Meier curves were calculated for visualizing the cumulative event-free survival of patients with normal and abnormal CMR for both endpoints. A log-rank test was performed to compare both survival curves. A multivariable Cox proportional hazard model was used for analyzing independent associations with cardiac mortality and the secondary endpoint. P-values <0.05 were considered significant. All p-values are results of two-tailed tests. Statistical analyses were performed using the SAS© statistical package, version 9.2 (SAS, Cary, North Carolina).

## Results

### Patient characteristics

Baseline characteristics are displayed in Table 1. Follow-up was available for n = 405 patients (all-comers, 54.8% in-patients, 38% referrals from cardiologists) (Figure 1). Median follow-up time was 1591 days. Most frequent symptoms or findings leading to CMR were angina/chest pain (53.6%), dyspnea (33.8%) and ECG abnormalities (32.3%) (several symptoms or findings could be present, thus these numbers do not add up to 100%). Viral prodromes, such as gastrointestinal or upper respiratory symptoms, were present in 32.1% of patients. Invasive coronary angiography was performed in 50.6% of the patients because CAD was suspected initially, including all patients older than 50 years and some younger patients with extensive cardiovascular risk profiles or ECG abnormalities suggesting coronary artery disease. CAD was detected in two of the included patients (0.5%), but both had no stenosis >50% of any large epicardial vessel (maximum was distal 70% RPLD-stenosis), and CAD was deemed not responsible for their clinical complaints (both heart failure).

**Table 1:** Baseline patient characteristics

All patients with follow-up	405 (92.5)
Time to follow-up [days]	1591 (1490–1739)
Gender, female	177 (43.7)
Age [years]	47.9 (36.9-60.8)
Referring physician	
Inpatients	222 (54.8)
Outpatients referred by cardiologists	154 (38.0)
Outpatients referred by general practitioners	29 (7.2)
Primary cardiac symptoms leading to CMR (multiple possible)	
Reduced LVEF	82 (20.2)
Pericardial effusion	6 (1.5)
ECG abnormality	131 (32.3)
Palpitations	92 (22.7)
Dyspnea	137 (33.8)
Angina/Chest pain	217 (53.6)
Abnormal fatigue	96 (23.8)
Wall motion abnormality	17 (4.2)
Ventricular arrythmias/Extrasystoles	45 (11.1)
Aborted SCD	6 (1.5)
Viral prodrome/history of infectious symptoms	130 (32.1)
Atrial fibrillation	50 (12.4)
Elevated troponin	38 (9.4)
Coronary angiography performed	205 (50.6)
EMB performed	78 (20.5)
Histopathological myocarditis in EMB	53 (68.8)
PVB19	29 (37.7)
HHV6	12 (15.6)
Double infection PVB19/HHV6	10 (13.0)
EBV	1 (1.3)
CMR imaging parameters	
LVEF [%]	62.5 (55.0-68.0)
LV-EDV [ml]	137 (110–164)
LV-ESV [ml]	50 (36–72)
LVEDD [mm]	50 (46.0-54.5)
Pericardial effusion present	76 (18.8)
LGE present	114 (28.3)
Final diagnosis based on CMR	
No cardiac pathology	225 (55.6)
Myocarditis	116 (28.8)
Other cardiac pathology	63 (15.6)

Values shown are n (%) or medians and IQR = interquartile range (25th-75th percentiles) *CMR*, cardiovascular magnetic resonance; *LVEF*, left ventricular ejection fraction; *SCD*, sudden cardiac death, *EMB*, endomyocardial biopsy, *PVB19*, parvovirus B 19; *HHV6*, human herpes virus 6; *EBV*, ebstein-barr virus; *CMR*, cardiovascular magnetic resonance; *EDV*, enddiastolic volume; *ESV*, endsystolic volume; *LVEDD*, left ventricular enddiastolic diameter; *LGE*, late gadolinium enhancement.

The CMR diagnosis “myocarditis” was made in 28.8%, “normal” in 55.6%, and “other cardiac pathology (e.g. DCM)” in 15.6% of patients. As our main aim was to demonstrate that patients with clinically suspected myocarditis and normal CMR have a good prognosis, the cohort was divided in those with normal CMR (55.6%), and those with abnormal CMR (44.4%, comprising CMR diagnosis of myocarditis, as well as other cardiac pathology). Clinically indicated EMB was performed in 20.5% of the patients, revealing myocarditis in 68.8% of cases, with PVB 19 as the most common virus.

All patients presenting with heart failure were started with heart failure treatment according to the guidelines applicable at that time [13], which was continued at the discretion of the individual patient’s cardiologist. If indicated, an ICD was offered, which was accepted by 14 patients. Twelve of these had an abnormal CMR, and 2 patients a normal CMR (one of those got an ICD for secondary prophylaxis following survived SCD as index event leading to CMR work-up, the other because an ion channel disease was diagnosed during follow-up).

### CMR findings

The median time between onset of symptoms and the CMR scan was 14 days, ranging from 1–70 days. Mean LV-EF of all patients was 62.5%, mean LV-EDV was 137 ml. LGE was present in 28.3% of the patients, pericardial effusion in 18.8%. A normal CMR (defined as LV-EF ≥ 60%, AND LV-EDV ≤ 180 ml, AND no LGE present) was found in 55.6% of patients.

**Figure 2:**
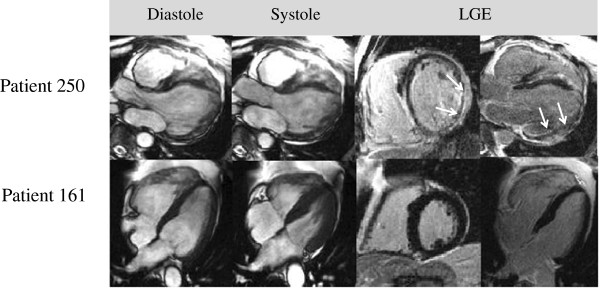
**Patients with similar symptoms, but different CMR results and outcomes. Patient 250** presented with dyspnea and chest pain, the same symptoms as **patient 161**. While in patient 250 CMR revealed an EF of 22% and epicardial LGE of the posterolateral wall typical for myocarditis (EMB: viral HHV6 myocarditis, no other pathology (e.g. no sarcoid)), patient 161 had a normal CMR. **Patient 250** died from SCD during follow-up while patient 161 had no events.

Dividing the patients in two groups (normal CMR vs. abnormal CMR) revealed several differences at baseline (Table 2): Patients with normal CMR were more often female (54.2% vs. 30.6%), and palpitations as the primary symptom leading to CMR was more frequent. Dyspnea as primary symptom was more frequent in patients with abnormal CMR (43.3 vs. 26.2%, p < 0.001), while chest pain was more often reported in patients with normal CMR (47.2 vs. 58.7%, p < 0.05). Figure 2 displays CMR results of two patients who presented with similar symptoms (i.e. dyspnea and chest pain) but had totally different findings in CMR and different clinical outcomes.

**Table 2:** Characteristics of patients with normal (no Pathology) and abnormal (any Pathology) CMR

	**CMR normal**	**CMR not normal**	**p-value**	**OR (95% - CI)**
	**(n = 225)**	**(n = 180)**		
Age [years]	46 (35.6-57.1)	49.7 (38.6-64.3)	<0.01	
Gender, female	122 (54.2)	55 (30.6)	<0.0001	2.69 (1.78-4.06)
Referring physician				
Inpatients	108 (48.0)	114 (63.3)	<0.01	0.56 (0.38-0.84)
Outpatients referred by cardiologists	98 (43.6)	56 (31.1)	<0.05	1.71 (1.13-2.58)
Outpatients referred by general practitioners	19 (8.4)	10 (5.6)	0.26	1.57 (0.71-3.46)
Primary cardiac symptoms leading to CMR				
Reduced LVEF	15 (6.7)	67 (37.2)	<0.0001	0.12 (0.07-0.22)
Pericardial effusion	4 (1.8)	2 (1.1)	0.58	1.61 (0.29-8.90)
ECG abnormality	77 (34.2)	54 (30.0)	0.37	1.21 (0.80-1.85)
Palpitations	66 (29.3)	26 (14.4)	<0.001	2.46 (1.48-4.07)
Dyspnea	59 (26.2)	78 (43.3)	<0.001	0.46 (0.31-0.71)
Angina/Chest pain	132 (58.7)	85 (47.2)	<0.05	1.59 (1.07-2.35)
Abnormal fatigue	57 (25.3)	39 (21.8)	0.41	1.22 (0.77-1.94)
Wall motion abnormality	8 (3.6)	9 (5.0)	0.47	0.70 (0.26-1.85)
Ventricular arrythmias/Extrasystoles	29 (12.9)	16 (8.9)	0.20	1.52 (0.80-2.89)
Aborted SCD	3 (1.3)	3 (1.7)	0.78	0.80 (0.16-4.00)
Viral prodrome/history of infectious symptoms	69 (30.7)	61 (33.9)	0.49	0.86 (0.57-1.31)
Atrial fibrillation	14 (6.2)	36 (20.1)	<0.0001	0.26 (0.14-0.51)
Elevated troponin	8(3.6)	30 (16.7)	<0.0001	0.18 (0.08-0.41)
EMB performed	12 (5.7)	66 (38.6)	<0.0001	0.10 (0.05-0.19)
Histopathological myocarditis in EMB	7 (58.3)	46 (70.8)	0.39	0.58 (0.16-2.05)
CMR imaging parameters				
LVEF [%]	66 (62.0-70.0)	54 (38.0-63.0)	<0.0001	
LV-EDV [ml]	122 (103–148)	160 (128–204)	<0.0001	
LV-ESV [ml]	42 (32.0-51.5)	72 (52–109)	<0.0001	
LVEDD [mm]	48 (44–52)	54 (49–59)	<0.0001	
Pericardial effusion present	0	76 (42.5)	<0.0001	
LGE present	0	114 (64.0)	<0.0001	
Symptoms at follow-up				
Angina pectoris	40 (19.8)	23 (15.0)	0.24	1.40 (0.80-2.45)
Other chest pain (non anginal)	17 (8.5)	15 (9.8)	0.66	0.85 (0.41-1.76)
Palpitations	34 (16.9)	22 (14.4)	0.52	1.21 (0.68-2.17)
NYHA class ≥ 2	53 (24.9)	58 (36.3)	<0.05	0.58 (0.37-0.91)
Medication				
Betablockers at follow-up	51 (24.4)	80 (49.1)	<0.0001	0.33 (0.22-0.52)
ACEI/ARB at follow-up	39 (18.7)	68 (41.7)	<0.0001	0.32 (0.20-0.51)
Events during follow-up				
Death	2 (0.9)	11 (6.1)	<0.01	0.14 (0.03-0.63)
Cardiac death	0	7 (63.6)	0.51	
Aborted SCD	0	1 (0.6)	0.25	
ICD shocks	0	2 (1.3)	0.10	
Hospitalization for heart failure	1 (0.5)	16 (9.5)	<0.0001	0.04 (0.01-0.34)

Values shown are n (%) or medians (25th–75th percentile). Abbreviations are the same as in Table 1.

**Figure 3:**
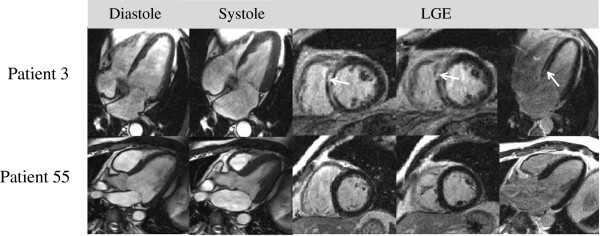
**Patients with histologically proven myocarditis, but different CMR results and outcomes. Patient 3** EMB demonstrated myocarditis with low copy numbers of PVB19. CMR revealed impaired ventricular function (LV-EF 36%) in an enlarged left ventricle (EDV 190 ml) and septal LGE. The patient suffered SCD during follow-up. **Patient 55** suffered from chest pain and abnormal fatigue, blood chemistry returned an elevated troponin. EMB revealed chronic myocarditis with intramyocardial presence of HHV6 and PVB19, but CMR was completely normal. This patient did not suffer any events and did not report any cardiac symptoms at follow-up.

There was no difference in the frequency of viral prodromes in the two groups (30.7 vs. 33.9%, p = 0.49). An elevated troponin was more likely in patients with abnormal CMR (16.7 vs. 3.6%, p < 0.0001), but not very frequent overall. Clinically indicated EMB was performed in 38.6% of the patients with abnormal CMR, and 5.7% of those with normal CMR. In the patients with normal CMR the clinical suspicion of myocarditis was that strong that EMB was done to verify the diagnosis in the absence of CMR findings. Myocarditis was diagnosed in the majority of all patients who underwent EMB (58.3% of those with normal CMR, and 70.8% of those with abnormal CMR). Parvovirus B19 was the most common virus, followed by human herpes virus 6. Note that independently of histopathological findings no patient with normal CMR suffered cardiac death or any major event, which is illustrated by Figure 3.

### Follow-up results

**Figure 4:**
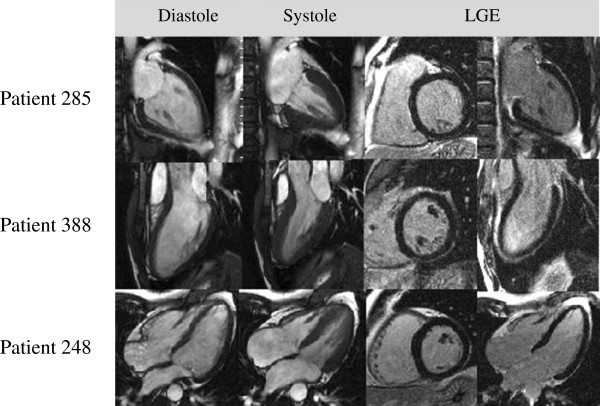
**CMR images of patients with different symptoms and histories suggestive of myocarditis. Patient 285**: 48-year-old female referred by a cardiologist for work-up of dyspnea, chest pain and abnormal fatigue, occurring after a viral infection. **Patient 388**: 28-year-old male who presented with palpitations, chest pain and abnormal fatigue after sinusitis. ECG showed ST-elevations suggestive of myocarditis. **Patient 248**: 49-year-old male, suffering from dyspnea, palpitations and ventricular extrasystoles following viral gastroenteritis. All these patients had normal CMR results, and in follow-up, there were no cardiac events in any of these patients. At follow-up, all were without any cardiac symptoms.

During follow-up 13 patients died, (11 of these with abnormal CMR and two patients with normal CMR), yielding an overall mortality of 3.2%. One of the two patients with normal CMR who died was 91 years old and died of major stroke, the other died at 61 years from bronchial carcinoma that had been accidentally diagnosed at index CMR. In the abnormal CMR group there were 10 major adverse cardiac events, including seven cardiac deaths, one aborted SCD and two cases of appropriate ICD shocks. In addition, four patients died from non-cardiac reasons (one car accident and three malignancies). Tables 3 and 4 demonstrate univariate analyses for the primary and secondary endpoints. We found no significant correlation between events and clinical symptoms leading to CMR, except for reduced ejection fraction (both endpoints) and dyspnea, which were significantly more frequent in patients with cardiac death or hospitalization for heart failure. Figure 4 displays CMR images of three patients who all presented with symptoms and histories typical for myocarditis, but had normal results on CMR; none of these patients suffered an event.

**Table 3:** Endpoint 1 – cardiac death, aborted SCD, appropriate ICD discharge

	**Endpoint 1**	**No endpoint**	**p-value**	**OR (95% - CI)**
	**(n = 10)**	**(n = 395)**		
Age [years]	66.1 (59.2-68.6)	47.7 (36.4-60.2)	<0.01	
Gender, female	4 (40)	173 (43.8)	0.81	0.86 (0.24-3.08)
Referring physician				
Inpatients	7(70)	215 (54.4)	0.31	1.99 (0.51-7.82)
Outpatients referred by cardiologists	3 (30)	151 (38.2)	0.60	0.69 (0.18-2.72)
Outpatients referred by general practitioners	0	29 (7.3)	0.37	
Primary cardiac symptoms leading to CMR				
Reduced LVEF	6 (60)	76 (19.2)	<0.01	6.30 (1.73-22.86)
Pericardial effusion	0 (0)	6 (1.5)	0.69	
ECG abnormality	3 (30)	128 (32.4)	0.87	0.89 (0.23-3.51)
Palpitations	1 (10)	91 (23)	0.33	0.37 (0.05-2.97)
Dyspnea	6 (60)	131 (33.2)	0.08	3.02 (0.84-10.90)
Angina/Chest pain	4 (40)	213 (53.9)	0.38	0.57 (0.16-2.05)
Abnormal fatigue	2 (20)	94 (23.9)	0.78	0.80 (0.17-3.82)
Wall motion abnormality	0	17 (4.3)	0.50	
Ventricular arrythmias/Extrasystoles	0	45 (11.4)	0.26	
Aborted SCD	0	6 (1.5)	0.69	
Viral prodrome/history of infectious symptoms	2 (20)	128 (32.4)	0.41	0.52 (0.11-2.49)
Atrial fibrillation	2 (20)	48 (12.2)	0.46	1.80 (0.37-8.74)
Elevated troponin	0	38 (9.6)	0.30	
EMB performed	6 (60)	72 (19.5)	<0.01	6.21 (1.71-22.58)
Histopathological myocarditis in EMB	4 (66.7)	49 (69)	0.91	0.90 (0.15-5.27)
CMR imaging parameters				
LVEF [%]	34.5 (22.0-43.0)	63.0 (56–69)	<0.0001	
LV-EDV [ml]	197 (144–269)	136 (110–163)	<0.01	
LV-ESV [ml]	127 (86–188)	49 (36–68)	<0.001	
LVEDD [mm]	61 (54–65)	50 (46–54)	<0.01	
Pericardial effusion present	6 (60)	70 (17.7)	<0.01	6.94 (1.91-25.25)
LGE present	8 (80.0)	106 (27)	<0.001	10.83 (2.26-51.82)
Final diagnosis based on CMR				
No cardiac pathology	0	225 (57)	<0.001	
Myocarditis	8 (80)	108 (27.3)	<0.001	10.63 (2.22-50.85)
Other cardiac pathology	2 (20)	61 (15.4)	0.69	1.37 (0.28-6.60)
Medication				
Betablockers at follow-up	6 (75)	125 (34.3)	<0.05	5.74 (1.14-28.84)
ACEI/ARB at follow-up	7 (87.5)	100 (27.5)	<0.001	18.48 (2.25-152.1)

Values shown are n (%) or medians (25th -75th percentiles) Abbreviations are the same as in Table 1.

**Table 4:** Endpoint 2 – death, aborted SCD, appropriate ICD discharge or hospitalization for heart failure

	**Endpoint 2**	**No endpoint**	**p-value**	**OR (95% - CI)**
	**(n = 26)**	**(n = 379)**		
Age [years]	61.1 (51.2-68.6)	47 (36–59.5)	<0.001	
Gender, female	9 (34.6)	168 (44.3)	0.33	0.66 (0.29-1.53)
Referring physician				
Inpatients	21 (80.8)	201 (53.0)	<0.01	3.80 (1.40-10.28)
Outpatients referred by cardiologists	5 (19.2)	149 (39.3)	<0.05	0.37 (0.14-0.99)
Outpatients referred by general practitioners	0	29 (7.7)	0.14	
Primary cardiac symptoms leading to CMR				
Reduced LVEF	15 (57.7)	67 (17.7)	<0.0001	6.35 (2.79-14.44)
Pericardial effusion	0	6 (1.6)	0.52	
ECG abnormality	7 (26.9)	124 (32.7)	0.54	0.76 (0.31-1.85)
Palpitations	5 (19.2)	87 (23)	0.66	0.80 (0.29-2.18)
Dyspnea	18 (69.2)	119 (31.4)	<0.0001	4.92 (2.08-11.62)
Angina/Chest pain	10 (38.5)	207 (54.6)	0.11	0.52 (0.23-1.17)
Abnormal fatigue	4 (15.4)	92 (24.3)	0.30	0.57 (0.19-1.68)
Wall motion abnormality	0	17 (4.5)	0.27	
Ventricular arrythmias/Extrasystoles	2 (7.7)	43 (11.3)	0.57	0.65 (0.15-2.85)
Aborted SCD	0	6 (1.6)	0.52	
Viral Prodrome/history of infectious symptoms	4 (15.4)	126 (33.2)	0.06	0.37 (0.05-2.81)
Atrial fibrillation	9 (34.6)	41 (10.8)	<0.001	4.35 (1.82-10.39)
Elevated troponin	1 (3.8)	37 (9.8)	0.32	0.37 (0.05-2.81)
EMB performed	16 (64)	62 (17.5)	<0.0001	8.40 (3.55-19.88)
Histological myocarditis in EMB	10 (62.5)	43 (70.5)	0.54	0.70 (0.22-2.21)
CMR imaging parameters				
LVEF [%]	39 (22.0-59.0)	63 (57.0-69.0)	<0.0001	
LV-EDV [ml]	181 (126.0-284.0)	136 (108.0-162.0)	<0.001	
LV-ESV [ml]	123 (52.0-188.0)	49 (36.0-67.0)	<0.0001	
LVEDD [mm]	58.5 (54.0-65.0)	50 (45.0-54.0)	<0.0001	
Pericardial effusion present	11 (42.3)	65 (17.2)	<0.01	3.53 (1.55-8.04)
LGE present	18 (69.2)	96 (25.5)	<0.0001	6.59 (2.77-15.63)
Final diagnosis based on CMR				
No cardiac pathology	1 (3.8)	224 (59.1)	<0.0001	6.59 (2.77-15.63)
Myocarditis	17 (65.4)	99 (26.1)	<0.0001	5.34 (2.31-12.37)
Other cardiac pathology	8 (30.8)	55 (14.5)	<0.05	2.62 (1.09-6.32)
Medication				
Betablockers at follow-up	20 (87.0)	111 (31.8)	<0.0001	14.29 (4.16-49.11)
ACEI/ARB at follow-up	20 (87.0)	87 (24.09)	<0.0001	20.08 (5.82-69.20)

Values shown are n (%) or medians (25th -75th percentiles) Abbreviations are the same as in Table 1.

Heart failure medication at follow-up was more common in patients with abnormal CMR (β-blocker in 49.1% vs. 24.4%, p < 0.0001, ACEI/ARB in 41.7% vs. 18.7%, <0.0001), and both β-blockers and ACE/ARB were taken frequently by patients suffering events (87% β-blocker and ACE/ARB in patients reaching endpoint 2).

All CMR parameters evaluated were significantly different between patients who had an event and those who did not. The odds ratio (OR) for the presence of LGE for the primary endpoint in univariate analyses was 10.83 (2.26-51.82), p <0.001.

**Figure 5:**
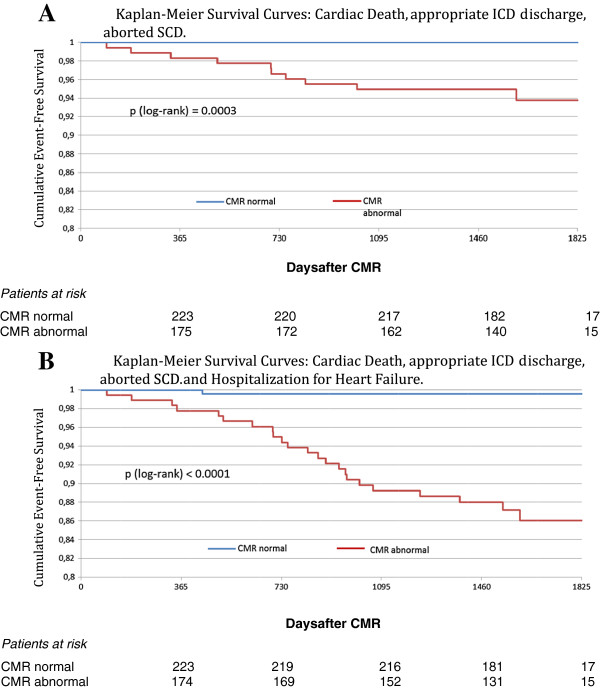
**Kaplan-Meier survival curves with regard to cardiac death, appropriate ICD discharge, aborted SCD (A), and cardiac death, appropriate ICD discharge, aborted SCD and hospitalization for heart failure (B).** The number of patients at risk is shown at the bottom of the figures. Abbreviations as in Table 1.

Kaplan-Meier analysis with log-rank test shows a significant difference for the primary endpoint (cardiac death, appropriate ICD discharge and aborted sudden cardiac death) between patients with normal and abnormal CMR (p = 0.0003), as well as for the secondary endpoint (endpoint 1 + hospitalization for heart failure, p < 0.0001). Kaplan-Meier curves can be viewed in Figure 5.

Multivariate Cox proportional hazards regression analysis including the presence of LGE, LV-EF and LV-EDV as measured by CMR revealed LV-EF as the best independent predictor of cardiac mortality (primary endpoint, hazard ratio [HR]: 0.939 per % increase, p = 0.01). In this model neither initial LV-EDV (HR: 0.999 per ml increase, p = 0.87) nor presence of LGE (HR: 3.98, p = 0.11) reached significance. Looking at endpoint 2, both LGE (HR: 2.919, p = 0.02), and LV-EF (HR: 0.965 per % increase, p = 0.03) were independent predictors, while LV-EDV (HR per ml increase 1.002, p = 0.33) did not reach significance.

## Discussion

This is the largest long-term follow-up dataset evaluating the prognostic value of clinical routine CMR in patients with clinically suspected myocarditis (n = 405). We found that a significant number of patients with abnormal CMR suffered major adverse cardiac events (cardiac death, ICD discharge, or aborted SCD, n = 10, 5.6% of all patients with abnormal CMR), while no patient with normal CMR had any major adverse cardiac event. Interestingly, only one patient with normal CMR suffered any cardiac event (one hospitalization for heart-failure only). Kaplan-Meier analysis with log-rank test confirmed a highly significant prognostic difference between patients with normal and abnormal CMR regarding major adverse and all adverse events.

### Patient characteristics

Most patients presented or were referred for work-up of chest pain, dyspnea, or ECG abnormalities, reflecting a much less symptomatic population in comparison to our previous reports [1,7,9]. This is also underscored by the fact that in the current population just 9.4% of patients had elevated troponin at presentation. However, types of viruses found are similar to our [1,7,9] and other previous reports [14,15].

### CMR findings

We found a broad range of normal or enlarged ventricles with either normal or impaired function. Two-hundred-twenty-five patients had a completely normal CMR according to our definition, whereas 180 patients had an abnormal CMR. LGE was present in 118 of the 180 abnormal patients, yielding a prevalence of 28.3% in the entire population, and was usually located in the subepicardial or intramural areas of the LV, which is in line with previous findings [7,9,16].

Interestingly, patients presenting with dyspnea as primary symptom had an abnormal CMR more frequently (43.3 vs. 26.2%, p < 0.001), while chest pain was more often reported by patients with normal CMR (47.2 vs. 58.7%, p < 0.05), supporting previous reports of a relation between symptoms, CMR findings and clinical outcome [9].

As described by Grün et al. [1] and others [17] our data also suggest that patients with scar demonstrated by LGE have larger ventricles and poorer LV-EF compared to those without scar (Table 2, abnormal CMR). As scarring may lead to LV dilatation and impaired LV-EF, this finding conceptually makes sense.

### Follow-up results

In our population of 405 consecutive patients presenting for CMR work-up of clinically suspected myocarditis overall mortality was 3.2%. This event rate is significantly lower that in our previous report [1] of patients with biopsy proven myocarditis (3.2% vs. 19.2%), also underscoring the differences in morbidity between the all comers presenting with mild symptoms in the present population, and patients with biopsy proven myocarditis previously reported.

In the group of 13 patients suffering death during follow-up, only two individuals with normal CMR died. As described above, those patients died from non-cardiac events (stroke and malignancy, see Results), whereas all other deaths occurred in the group of patients with abnormal CMR, and the majority was caused by cardiac events. Importantly, no patient with normal CMR suffered cardiac death, or any other major adverse cardiac event (Figure 5 upper panel). Only one patient with normal CMR was hospitalized for heart failure during follow-up, emphasizing the prognostic impact of a normal CMR in consecutive patients with clinically suspected myocarditis.

Multivariate Cox analyses revealed LV-EF as the best independent predictor of cardiac death in our group of patients with suspected myocarditis, which is somewhat discrepant to previous results [1,17]. This finding is most likely explained by the low prevalence of LGE in the current population (28.3% vs. 53.2% in the cohort reported by Grün), and the lower rate of adverse events (3.2% vs. 19.2%). Thus, it seems very likely, especially with regard to the results of the univariate analysis described above (OR for the presence of LGE for the primary endpoint 10.83, p < 0.001), that LGE (current multivariate HR: 3.98, p = 0.11) would turn out to be significant in a larger group with more events. This is also supported by the fact that for endpoint 2 (including hospitalization for heart failure) both presence of LGE (HR: 2.919, p = 0.02), and LV-EF (HR: 0.965, p = 0.03) were independent predictors of events.

Nevertheless, it is very important to keep in mind that the main aim of this study was not to identify predictors of adverse events in patients with suspected myocarditis, but to establish clinical routine risk stratification of patients with suspected myocarditis by demonstrating that adverse cardiac events are extremely rare in patients with normal CMR, independent of their clinical symptoms.

### Clinical implications

Although our data reveal a strong association between a normal CMR and a good long-term prognosis for consecutive patients presenting for CMR work-up of clinically suspected myocarditis, prospectively designed international trials and/or registries are required to definitively establish CMR risk stratification in this specific setting.

However, with regard to our current and previous data [1] some recommendations for clinical management seem appropriate: 1) The present data indicate that patients with clinically suspected myocarditis and normal CMR have an excellent long-term prognosis. Although CMR certainly cannot always diagnose myocarditis (e.g. histopathological evidence of myocarditis in some patients with normal CMR, who underwent clinically indicated EMB, see Results), it seems to be very effective in excluding relevant cardiac pathology leading to adverse cardiac events. Thus, it represents a powerful non-invasive tool for risk stratification in patients with clinically suspected or known myocarditis, which can give suffering patients and worrying physicians some peace of mind independent of the clinical symptoms and other findings. 2) As in previous datasets [1,9] we identified impaired LV-EF and symptoms of heart failure as important predictors of adverse cardiac events. This reproducible finding once more suggests that one should carefully optimize heart failure therapy in all patients with suspected or known myocarditis presenting with even the mildest physical signs of heart failure.

However, as β-blockers were taken by most of the patients suffering an adverse cardiac event, a protective effect as suggested by other authors [14] cannot be derived from our current data, although such an effect cannot be excluded.

### Limitations

There may be a referral bias in the current population, resulting in the inclusion of more healthy people. However, this seems not very likely since there was no significant difference between the groups of inpatients, referrals from cardiologists, and referrals from general practitioners in reaching endpoint 1 vs. no endpoint (see Table 3).

In addition, it might be criticized that EMB was performed in only 20.5% of the current patients; while in other important studies focusing on prognosis in myocarditis all patients underwent EMB [1,14,18,19]. However, according to current guidelines, EMB is not commonly indicated [8]. Especially in patients without any heart failure, or with late onset (> 4 weeks) the risks of the procedure (including perforation, pericardial tamponade, ventricular arrhythmias, embolization and others) may outweigh the possible benefits. Based on our study design (all comers with clinically suspected myocarditis), most of our patients did not have an indication for EMB. Nevertheless, all had symptoms suggestive of myocarditis leading to presentation at a cardiologist or hospital admittance, and although CMR certainly cannot always diagnose myocarditis [20] it seems to be very effective in excluding relevant cardiac pathology leading to adverse cardiac events.

## Conclusions

In our unselected population of consecutive patients referred for CMR work-up of clinically suspected myocarditis, patients with normal CMR (according to our definition) have a good prognosis, independent of their clinical symptoms and other findings.

Thus, CMR represents a powerful non-invasive tool to identify patients with clinically suspected myocarditis who are at low risk for future events and hence may benefit from advice to getting back to normal life soon after symptoms abate.

## Abbreviations

CAD: Coronary artery disease; CMR: Cardiovascular magnetic resonance; ECG: Electrocardiogram; EMB: Endomyocardial biopsy; ICD: Implantable cardioverter-defibrillator; LV-EDV: Left ventricular end-diastolic volume; LV-EF: Left ventricular ejection fraction; HR: Hazard ratio; IQR: Inter quartile range; LGE: Late gadolinium enhancement; LV: Left ventricle; OR: Odds ratio; SCD: Sudden cardiac death.

## Competing interests

The authors declare that they have no competing interest.

## Authors’ contributions

JS and SG contributed equally to the idea and design of the study, acquired and analyzed the data, and wrote the report. AW, SG, PO, KB, KK, RK, OB, SS contributed to the idea and design of the study, analysis of the data, and revision of the report. US contributed to the idea and design of the study, acquisition and analysis of the data, and revision of the report. HM designed the study, contributed to the acquisition and analysis of the data, and wrote the report. All authors read and approved the final manuscript.

## References

[B1] Grün S Schumm J Greulich S Wagner A Schneider S Bruder O Kispert EM Hill S Ong P Klingel K Kandolf R Sechtem U Mahrholdt H Long-term follow-up of biopsy-proven viral myocarditis: predictors of mortality and incomplete recovery J Am Coll Cardiol 2012 59 1604 1615 10.1016/j.jacc.2012.01.007 22365425

[B2] Fabre A Sheppard MN Sudden adult death syndrome and other non-ischaemic causes of sudden cardiac death Heart 2006 92 316 320 15923280 10.1136/hrt.2004.045518 PMC1860827

[B3] Bruder O Wagner A Jensen CJ Schneider S Ong P Kispert EM Nassenstein K Schlosser T Sabin GV Sechtem U Mahrholdt H Myocardial scar visualized by cardiovascular magnetic resonance imaging predicts major adverse events in patients with hypertrophic cardiomyopathy J Am Coll Cardiol 2010 56 875 887 10.1016/j.jacc.2010.05.007 20667520

[B4] Bingham SE Hachamovitch R Incremental prognostic significance of combined cardiac magnetic resonance imaging, adenosine stress perfusion, delayed enhancement, and left ventricular function over preimaging information for the prediction of adverse events Circulation 2011 123 1509 1518 10.1161/CIRCULATIONAHA.109.907659 21444886

[B5] Gulati A Jabbour A Ismail TF Guha K Khwaja J Raza S Morarji K Brown TD Ismail NA Dweck MR Di Pietro E Roughton M Wage R Daryani Y O’Hanlon R Sheppard MN Alpendurada F Lyon AR Cook SA Cowie MR Assomull RG Pennell DJ Prasad SK Association of fibrosis with mortality and sudden cardiac death in patients with nonischemic dilated cardiomyopathy JAMA 2013 309 896 908 10.1001/jama.2013.1363 23462786

[B6] Greulich S Deluigi CC Gloekler S Wahl A Zürn C Kramer U Nothnagel D Bültel H Schumm J Grün S Ong P Wagner A Schneider S Nassenstein K Gawaz M Sechtem U Bruder O Mahrholdt H CMR imaging predicts death and other adverse events in suspected cardiac sarcoidosis JACC Cardiovasc Imaging 2013 6 501 511 10.1016/j.jcmg.2012.10.021 23498675

[B7] Mahrholdt H Goedecke C Wagner A Meinhardt G Athanasiadis A Vogelsberg H Fritz P Klingel K Kandolf R Sechtem U Cardiovascular magnetic resonance assessment of human myocarditis: a comparison to histology and molecular pathology Circulation 2004 109 1250 1258 10.1161/01.CIR.0000118493.13323.81 14993139

[B8] Cooper LT Baughman KL Feldman AM Frustaci A Jessup M Kuhl U Levine GN Narula J Starling RC Towbin J Virmani R American Heart Association The role of endomyocardial biopsy in the management of cardiovascular disease: a scientific statement from the American Heart Association, the American College of Cardiology, and the European Society of Cardiology Circulation 2007 116 2216 2233 10.1161/CIRCULATIONAHA.107.186093 17959655

[B9] Mahrholdt H Wagner A Deluigi CC Kispert E Hager S Meinhardt G Vogelsberg H Fritz P Dippon J Bock CT Klingel K Kandolf R Sechtem U Presentation, patterns of myocardial damage, and clinical course of viral myocarditis Circulation 2006 114 1581 1590 10.1161/CIRCULATIONAHA.105.606509 17015795

[B10] Kramer CM Barkhausen J Flamm SD Kim RJ Nagel E Standardized cardiovascular magnetic resonance imaging (CMR) protocols, society for cardiovascular magnetic resonance: board of trustees task force on standardized protocols J Cardiov Magn Reson 2008 10 35 10.1186/1532-429X-10-35 PMC2467420 18605997

[B11] Simonetti OP Kim RJ Fieno DS Hillenbrand HB Wu E Bundy JM Finn JP Judd RM An improved MR imaging technique for the visualization of myocardial infarction Radiology 2001 218 215 223 10.1148/radiology.218.1.r01ja50215 11152805

[B12] Mahrholdt H Wagner A Holly TA Elliott MD Bonow RO Kim RJ Judd RM Reproducibility of chronic infarct size measurement by contrast-enhanced magnetic resonance imaging Circulation 2002 106 2322 2327 10.1161/01.CIR.0000036368.63317.1C 12403661

[B13] Dickstein K Cohen-Solal A Filippatos G McMurray JJ Ponikowski P Poole-Wilson PA Strömberg A van Veldhuisen DJ Atar D Hoes AW Keren A Mebazaa A Nieminen M Priori SG Swedberg K ESC Committee for Practice Guidelines (CPG) ESC guidelines for the diagnosis and treatment of acute and chronic heart failure 2008: the Task Force for the diagnosis and treatment of acute and chronic heart failure 2008 of the European Society of Cardiology. Developed in collaboration with the Heart Failure Association of the ESC (HFA) and endorsed by the European Society of Intensive Care Medicine (ESICM) Eur J Heart Fail 2008 10 933 989 10.1016/j.ejheart.2008.08.005 18826876

[B14] Kindermann I Kindermann M Kandolf R Klingel K Bültmann B Müller T Lindinger A Böhm M Predictors of outcome in patients with suspected myocarditis Circulation 2008 118 639 648 Erratum in: *Circulation.* 2008 Sep 16; **118:** e493 10.1161/CIRCULATIONAHA.108.769489 18645053

[B15] Kühl U Pauschinger M Seeberg B Lassner D Noutsias M Poller W Schultheiss HP Viral persistence in the myocardium is associated with progressive cardiac dysfunction Circulation 2005 112 1965 1970 10.1161/CIRCULATIONAHA.105.548156 16172268

[B16] De Cobelli F Pieroni M Esposito A Chimenti C Belloni E Mellone R Canu T Perseghin G Gaudio C Maseri A Frustaci A Del Maschio A Delayed gadolinium-enhanced cardiac magnetic resonance in patients with chronic myocarditis presenting with heart failure or recurrent arrhythmias J Am Coll Cardiol 2006 47 1649 1654 10.1016/j.jacc.2005.11.067 16631005

[B17] Assomull RG Prasad SK Lyne J Smith G Burman ED Khan M Sheppard MN Poole-Wilson PA Pennell DJ Cardiovascular magnetic resonance, fibrosis, and prognosis in dilated cardiomyopathy J Am Coll Cardiol 2006 48 1977 1985 10.1016/j.jacc.2006.07.049 17112987

[B18] Ukena C Mahfoud F Kindermann I Kandolf R Kindermann M Böhm M Prognostic electrocardiographic parameters in patients with suspected myocarditis Eur J Heart Fail 2011 13 398 405 10.1093/eurjhf/hfq229 21239404

[B19] Escher F Westermann D Gaub R Pronk J Bock T Al-Saadi N Kühl U Schultheiss HP Tschöpe C Development of diastolic heart failure in a 6-year follow-up study in patients after acute myocarditis Heart 2011 97 709 714 10.1136/hrt.2010.199489 21134904

[B20] Abdel-Aty H Boyé P Zagrosek A Wassmuth R Kumar A Messroghli D Bock P Dietz R Friedrich MG Schulz-Menger J Diagnostic performance of cardiovascular magnetic resonance in patients with suspected acute myocarditis: comparison of different approaches J Am Coll Cardiol 2005 45 1815 1822 10.1016/j.jacc.2004.11.069 15936612

